# Enhancing predictive accuracy of the cardiac risk score in open abdominal aortic surgery: the role of left ventricular wall motion abnormalities

**DOI:** 10.3389/fcvm.2023.1239153

**Published:** 2023-12-01

**Authors:** Ivana Djokic, Biljana Milicic, Predrag Matic, Nenad Ilijevski, Milan Milojevic, Miomir Jovic

**Affiliations:** ^1^ Clinic for Anesthesia and Intensive Care, Dedinje Cardiovascular Institute, Belgrade, Serbia; ^2^ Department of Medical Statistics and Informatics, Faculty of Dental Medicine, University of Belgrade, Belgrade, Serbia; ^3^ Clinic for Vascular Surgery, Dedinje Cardiovascular Institute, Belgrade, Serbia; ^4^ School of Medicine, Belgrade University, Belgrade, Serbia; ^5^ Department of Cardiovascular Research, Dedinje Cardiovascular Institute, Belgrade, Serbia

**Keywords:** cardiac risk, open abdominal aortic surgery, left ventricular function, wall motion abnormalities, cardiac risk scores, VSG-CRI, Vascular Study Group Cardiac Risk Index

## Abstract

**Background:**

Open abdominal aortic surgery carries many potential complications, with cardiac adverse events being the most significant concern. The Vascular Study Group Cardiac Risk Index (VSG-CRI) is a commonly used tool for predicting severe cardiac complications and guiding clinical decision-making. However, despite the potential prognostic significance of left ventricular wall motion abnormalities (LVWMAs) and reduced LV ejection fraction (LVEF) for adverse outcomes, the VSG-CRI model has not accounted for them. Hence, the main objective of this study was to analyze the added value of LV wall motion on the discriminatory power of the modified VSG-CRI in predicting major postoperative cardiac complications.

**Methods:**

A prospective study was conducted involving 271 patients who underwent elective abdominal aortic surgery between 2019 and 2021. VSG-CRI scores were calculated, and preoperative transthoracic echocardiography was conducted for all patients. Subsequently, a modified version of the VSG-CRI, accounting for reduced LVEF and LVWMAs, was developed and incorporated into the dataset. The postoperative incidence of the composite endpoint of major adverse cardiac events (MACEs), including myocardial infarction, clinically relevant arrhythmias treated with medicaments or by cardioversion, or congestive heart failure, was assessed at discharge from the index hospitalization, with adjudicators blinded to events. The predictive accuracy of both the original and modified VSG-CRI was assessed using C-Statistics.

**Results:**

In total, 61 patients (22.5%) experienced MACEs. Among these patients, a significantly higher proportion had preoperative LVWMAs compared to those without (62.3% vs. 32.9%, *p* < 0.001). Multivariable regression analysis revealed the VSG-CRI [odds ratio (OR) 1.46, 95% confidence interval (CI) 1.21–1.77; *p* < 0.001] and LVWMA (OR 2.76; 95% CI 1.46–5.23; *p* = 0.002) as independent predictors of MACEs. Additionally, the modified VSG-CRI model demonstrated superior predictability compared to the baseline VSG-CRI model, suggesting an improved predictive performance for anticipating MACEs following abdominal aortic surgery [area under the curve (AUC) 0.74; 95% CI 0.68–0.81 vs. AUC 0.70; 95% CI 0.63–0.77; respectively].

**Conclusion:**

The findings of this study suggest that incorporating preoperative echocardiography can enhance the predictive accuracy of the VSG-CRI for predicting MACEs after open abdominal aortic surgery. Before its implementation in clinical settings, external validation is necessary to confirm the generalizability of this newly developed predictive model across different populations.

## 1. Introduction

Patients with vascular conditions often have co-existing forms of acquired cardiac disease. Consequently, they are exposed to a substantial risk of perioperative morbidity and mortality during elective vascular procedures (1). Therefore, predicting the cardiac risks of patients undergoing vascular surgery can guide the choice of treatment modalities, potentially lower perioperative morbidity and mortality, shorten hospital stays, and reduce healthcare costs (2). Numerous studies have extensively explored the associations of patient characteristics and comorbidities with the incidence of cardiac adverse events in noncardiac surgery (1, 3). As a result, multiple predictive risk models have been developed and introduced in daily practice over the years (4–7). Among these, the Vascular Study Group of New England Cardiac Risk Index (VSG-CRI) has emerged as the standard tool for predicting major adverse cardiac events (MACEs) in vascular surgery (8). The accuracy of VSG-CRI is attributed to its derivation from a large group of patients undergoing vascular surgery and its external validation (9). Aortic surgery is classified as high-risk and associated with a substantial likelihood of a significant cardiac event or death, with an estimated risk exceeding 5% (10, 11).

Major vascular surgery may lead to adverse cardiac events through various mechanisms. Among the most significant is clamping and unclamping the abdominal aorta during reconstructive surgery, which markedly impacts left ventricular (LV) pressure and volume loading. These alterations affect coronary perfusion and potentially contribute to cardiac complications, especially in those with co-existing cardiac conditions. Several studies have suggested that patients with cardiac wall motion abnormalities are at an increased risk of adverse events (12, 13). Additionally, many authors have underscored the value of preoperative transthoracic echocardiography (TTE) in predicting these events and guiding clinical decisions, especially by focusing on LV wall motion and LV ejection fraction (LVEF) to enhance patient outcomes (14, 15). Despite the correlation between lower LVEF, LV wall motion abnormalities (LVWMAs), and cardiac complications, the role of TTE parameters in predicting postoperative adverse events remains unclear (16). Although the VSG-CRI has demonstrated accuracy in predicting MACEs in vascular surgery, it may underestimate cardiac risk in patients with co-existing cardiac conditions, as cardiac imaging findings are not considered (17). Therefore, our study primarily aimed to investigate the added value of incorporating preoperative LVEF and LVWMAs findings to enhance the discriminatory power of the VSG-CRI score for predicting MACEs in patients undergoing abdominal aortic surgery.

## 2. Methods

### 2.1. Study population

This prospective observational study was conducted at Dedinje Cardiovascular Institute, a tertiary referral center, in collaboration with the Clinics for Anesthesia and Vascular Surgery. The study involved 271 consecutive patients scheduled for elective abdominal aortic surgery between October 2019 and September 2021. Of these, 200 patients underwent open infrarenal abdominal aortic aneurysm repair (OAAA), while 71 patients underwent open reconstructive aortic surgery for chronic aortoiliac occlusive disease. Inclusion criteria encompassed patients scheduled for elective OAAA as well as those requiring aortofemoral or aortoiliac bypass reconstruction due to aortoiliac occlusive disease. However, patients with surgical emergencies, ruptured abdominal aortic aneurysms, and those undergoing endovascular aortic aneurysm repair were excluded.

Compliance with the Declaration of Helsinki was ensured during the entire course of the research, and approval was granted by the Institutional Review Board of the Dedinje Cardiovascular Institute (approval code 3336) and the Ethics Committee of the Faculty of Medicine, University of Belgrade (approval code 1550/IX-9). Informed consent was obtained from all patients who participated in the study.

### 2.2. Perioperative assessment and development of risk score models

A comprehensive database was established during the preoperative period, with particular attention to data necessary for calculating the VSG-CRI score (8). This score considers patient characteristics such as age, coronary artery disease (CAD), congestive heart failure (CHF), chronic obstructive pulmonary disease, insulin-dependent diabetes mellitus, creatinine levels, smoking habits, long-term β-blockade therapy, previous coronary artery bypass grafting (CABG), or percutaneous coronary intervention (PCI). Information was obtained from anamnesis, medical records, and hospital examinations if needed. The VSG-CRI score was determined for all studied patients, ensuring no missing data, and validated within the same patient group.

Prior to the operation, each patient underwent a standard two-dimensional TTE examination to evaluate LVEF and the kinetics of the LV regional walls. These evaluations were conducted by a specialist imaging cardiologist from our Department of Echocardiography using a standardized assessment approach, ensuring precision in capturing the heart's structural movements and decreasing interobserver variability. Hypokinesia, dyskinesia, or akinesia of the LV walls were defined as LVWMAs. Reduced LVEF was defined as systolic EF < 40%, according to the recommendations from the European Society of Cardiology (ESC) Guidelines for diagnosis and treatment of heart failure (18). The newly acquired variables, LVWMA and reduced LVEF, were classified as binary, either “yes” or “no.” Subsequently, the VSG-CRI score was adopted to include any LVWMA and LVEF <40% as categorical variables, resulting in the development of the Modified VSG-CRI (M VSG-CRI) model.

### 2.3. Study endpoints

The primary endpoint of the present study was the incidence of the composite endpoint of MACEs, the outcome used in developing and validating the original VSG-CRI model (8). These individual MACE components included myocardial infarction (MI), clinically relevant arrhythmias treated with medicaments or by cardioversion, or CHF during the hospital stay. MI was defined by the presence of new changes in ST and T wave, elevated high-sensitivity troponin I levels, or documented evidence of a new wall motion abnormality from an echocardiogram. Significant arrhythmias were defined as those requiring therapeutic intervention, including medical or cardioversion. CHF was clinically diagnosed as pulmonary edema, confirmed through radiography and echocardiography findings and requiring prolonged treatment or readmission to the intensive care unit.

### 2.4. Statistical analysis

Data are presented using descriptive statistics, including central tendency and variability measures. Pearson's chi-square and Mann–Whitney tests were used to determine statistically significant differences between patients with and without MACEs for categorical and numerical variables, respectively. Univariable logistic regression analysis was conducted to verify the validity of potential candidate variables for building the subsequent multivariable model. Variance inflation factor (VIF) values were computed to assess multicollinearity among variables, with a VIF exceeding 5 indicating a significant correlation. Pearson's correlation coefficients were also determined, with a value less than 0.7 between two independent variables indicating no multicollinearity. Every correlation coefficient between variable pairs was less than 0.7, and the VIF values were between 1 and 2, suggesting but not entirely excluding collinearity among the independent variables. Multivariable logistic regression analysis was carried out using the stepwise backward conditional selection method with a 0.05 probability for both entry and removal. The odds ratio (OR), along with the 95% confidence interval (CI), were calculated for both the univariable and final multivariable regression analysis model. Subsequently, a modified score was calculated for the entire dataset, and the discriminatory capacity of both the baseline and modified VSG-CRI was examined using receiver operating characteristic curve analysis. The area under the curve (AUC), along with the 95% CI, was computed. A *p*-value of less than 0.05 was considered statistically significant. All data processing was conducted using SPSS statistical software ver. 25.0 (IBM, Armonk, NY, USA).

## 3. Results

### 3.1. Baseline and operative characteristics of patients

A total of 271 patients, predominantly male (86.7%), with a mean age of 65.9 ± 6.8 years, who underwent elective abdominal aortic surgery were included in this study. Among the patients, 80.8% were smokers and 47.2% had CAD (Table 1). Most surgeries (73.8%) were performed for OAAA, while the remaining procedures were performed for aortoiliac or aortofemoral reconstruction (26.2%).

**Table 1:** Baseline characteristics of the entire cohort, patients who experienced postoperative adverse cardiac events, and those who did not.

	Entire cohort (*N* = 271)	No MACE (*n* = 210)	MACE (*n* = 61)	*p*-value
Male	235 (86.7%)	180 (85.7%)	55 (90.2%)	0.37
Age, years	65.9 ± 6.8	65.4 ± 7.1	67.5 ± 5.5	0.073
Age <60 years	46 (17.0%)	43 (20.5%)	3 (4.9%)	0.034
60–69 years	143 (52.8%)	104 (49.5%)	39 (63.9%)	
70–79 years	77 (28.4%)	59 (28.1%)	18 (29.5%)	
≥80 years	5 (1.8%)	4 (1.9%)	1 (1.6%)	
Smoking history	219 (80.8%)	166 (79.0%)	53 (86.9%)	0.17
Coronary artery disease	128 (47.2%)	83 (39.5%)	45 (73.8%)	<0.001
CHF	11 (4.1%)	6 (2.9%)	5 (8.2%)	0.063
COPD	34 (12.5%)	19 (9.0%)	15 (24.6%)	0.001
Creatinine ≥1.8 mg/dl	15 (5.5%)	12 (5.7%)	3 (4.9%)	0.81
Insulin-dependent DM	16 (5.9%)	11 (5.2%)	5 (8.2%)	0.39
Prior PCI	58 (21.4%)	37 (17.6%)	21 (34.4%)	0.005
Prior CABG	39 (14.4%)	24 (11.4%)	15 (24.6%)	0.010
VSG-CRI score	4.5 ± 1.8	4.2 ± 1.8	5.5 ± 1.7	<0.001
ACEI/ARBs	181 (66.8%)	137 (65.2%)	44 (72.1%)	0.407
Chronic β-blockers	167 (61.6%)	127 (60.5%)	40 (65.6%)	0.471
Calcium channel blockers	104 (38.4%)	76 (36.2%)	28 (46%)	0.170
Diuretic, any	71 (26.2%)	49 (23.3%)	22 (36.1%)	0.124
Statins	170 (62.7%)	130 (62%)	40 (65.6%)	0.322
Antiplatelet medication, any	197 (72.7%)	150 (71.4%)	47 (77%)	0.386

Values are presented as either *n* (%) or mean ± SD, as appropriate. Statistical comparisons were made between the MACE and non-MACE subgroups.

ACEI, angiotensin-converting enzyme inhibitor; ARB, angiotensin receptor blocker; CABG, coronary artery bypass grafting; CHF, congestive heart failure; COPD, chronic obstructive pulmonary disease; DM, diabetes mellitus; MACE, major adverse cardiac event, a composite endpoint of myocardial infarction, significant arrhythmias treated either by medical therapies or cardioversion, or CHF during the hospital stay; PCI, percutaneous coronary intervention; SD, standard deviation; VSG-CRI, Vascular Study Group Cardiac Risk Index.

### 3.2. Incidence of MACE and its correlation with preoperative echocardiographic findings

Upon hospital discharge, 61 patients (22.5%) experienced MACEs. Of these, arrhythmias occurred in 53 patients (19.6%), including supraventricular, ventricular, and both entities in 8.5%, 9.2%, and 1.8%, respectively, and asystole in 0.7%, while five patients (1.8%) experienced MI. Nine patients (3.3%) developed postoperative CHF. Patients with a history of CAD were more likely to develop MACE (73.8% vs. 39.5%, *p* < 0.001). Those who experienced MACEs also had a higher mean VSG-CRI score of 5.5 ± 1.7 compared to 4.2 ± 1.8 for those who did not experience MACEs (*p* < 0.001) (Table 1).

According to the preoperative TTE assessment, 107 patients (39.5%) had LVWMAs, and 13 patients (5%) had LVEF < 40%. Among those who developed MACEs, there was a significantly higher proportion of patients with preoperative LVWMAs than those without MACEs (62.3% vs. 32.9%; *p* < 0.001). Furthermore, patients with segmental abnormalities in the lateral, posterior, septal, and inferior LV walls were notably more prevalent in the group that experienced postoperative MACEs, as detailed in Table 2. Additionally, patients who developed MACEs postoperatively significantly more often presented with an LVEF < 40% (11.5% vs. 2.9%, *p* = 0.006) (Table 2).

**Table 2:** Preoperative echocardiographic parameters of patients stratified by the presence or absence of postoperative major cardiac events.

	No MACE (*n* = 210)	MACE (*n* = 61)	*p*-value
LVWMA, any	69 (32.9%)	38 (62.3%)	<0.001
LVWMAs of the anterior wall	6 (2.9%)	2 (3.3%)	0.86
LVWMAs of the lateral wall	12 (5.7%)	13 (21.3%)	<0.001
LVWMAs of the posterior wall	44 (21%)	26 (42.6%)	0.001
LVWMAs of the septal wall	32 (15.2%)	17 (27.9%)	0.024
LVWMAs of the inferior wall	63 (30.0%)	36 (59.0%)	<0.001
LVEF < 40%	6 (2.9%)	7 (11.5%)	0.006

Values are presented as *n* (%).

LVWMAs, left ventricular wall motion abnormalities; LVEF, left ventricular ejection fraction; MACE, major adverse cardiac event, a composite endpoint of myocardial infarction, significant arrhythmias treated either by medical therapies or cardioversion, or congestive heart failure during the hospital stay.

### 3.3. Univariable and multivariable logistic regression analysis

Univariable analysis revealed the VSG-CRI, LVEF < 40%, and LVWMA as MACE predictors. However, the multivariable analysis only recognized the VSG-CRI (OR 1.46, 95% CI 1.21–1.77; *p* < 0.001) and LVWMA (OR 2.76, 95% CI 1.46–5.23; *p* = 0.002) as independent predictors, omitting LVEF < 40% (OR 1.51, 95% CI 0.44–5.20; *p* = 0.51) (Table 3). Patients with preoperative LVWMA were 2.76 times more likely to experience MACEs compared to those without LVWMA. Given the weighting coefficient for exponent B of 2.76, a value of three was assigned to the VSG-CRI score when LVWMAs were present, thus creating an M VSG-CRI. A higher mean M VSG-CRI score was observed in patients who developed MACEs than those without MACEs (7.4 ± 2.2 vs. 5.2 ± 2.4, *p* < 0.001; respectively).

**Table 3:** Determinants of MACEs following abdominal aortic surgery.

	Univariable OR (95% CI), *p*-value		Multivariable OR (95% CI), *p*-value	
VSG-CRI score	1.51 (1.27–1.81)	<0.001	1.46 (1.21–1.77)	<0.001
LVWMA, any	3.38 (1.87–6.11)	<0.001	2.76 (1.46–5.23)	0.002
LVEF < 40%	4.41 (1.42–13.66)	0.010	1.51 (0.44–5.20)	0.51

CI, confidence interval; LVEF, left ventricular ejection fraction; LVWMA, left ventricular wall motion abnormality; MACE, major adverse cardiac event, a composite endpoint of myocardial infarction, significant arrhythmias treated either by medical therapies or cardioversion, or congestive heart failure during the hospital stay; OR, odds ratio; VSG-CRI, Vascular Study Group Cardiac Risk Index.

### 3.4. The discriminatory ability of VSG-CRI and M VSG-CRI in predicting MACEs

**Figure 1:**
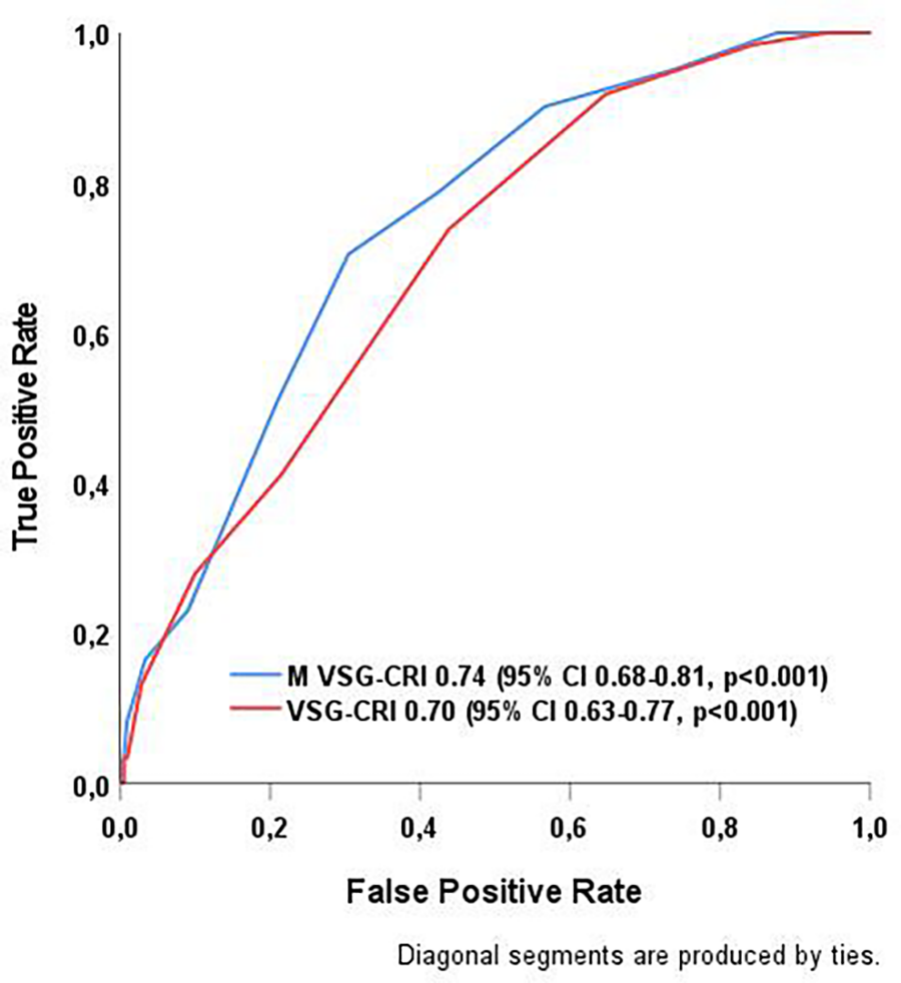
Comparison of the discriminatory capabilities of VSG-CRI and M VSG-CRI in predicting MACEs after abdominal aortic surgery. MACE, major adverse cardiac event, a composite endpoint of myocardial infarction, significant arrhythmias treated either by medical therapies or cardioversion, or congestive heart failure (CHF) during the hospital stay; ROC curve, receiver operating characteristic curve; VSG-CRI, Vascular Study Group Cardiac Risk Index. The Modified VSG-CRI (M VSG-CRI) additionally accounts for left ventricular wall motion abnormalities.

The VSG-CRI showed good discriminatory performance, with an AUC of 0.70 (95% CI 0.63–0.77; *p* < 0.001). In contrast, the M VSG-CRI showed enhanced discriminatory ability, with an AUC of 0.74 (95% CI 0.68–0.81; *p* < 0.001), as displayed in Figure 1.

### 3.5. VSG-CRI and M VSG-CRI as categorical variables

**Figure 2:**
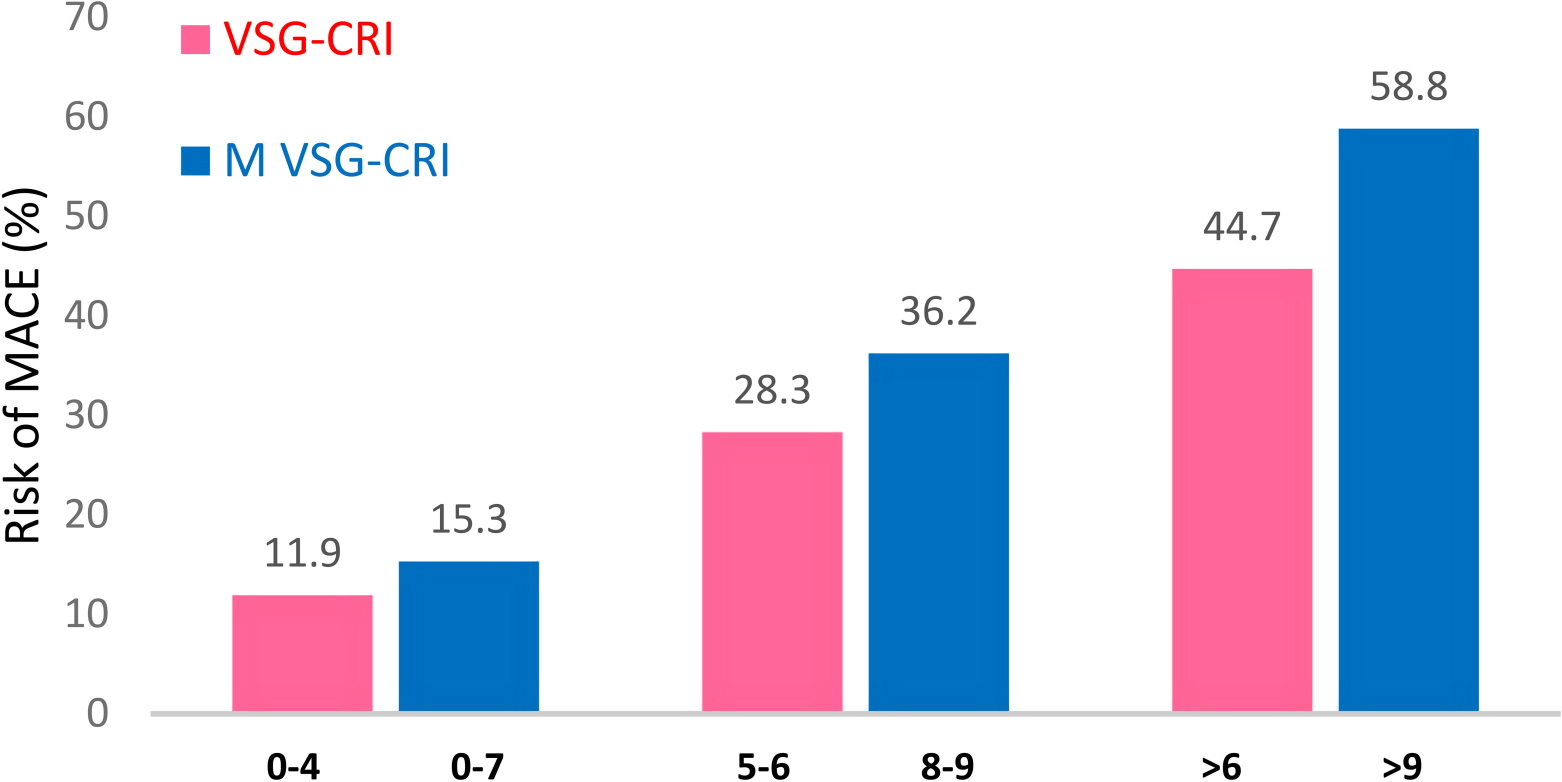
Distribution of patients experiencing MACEs according to the original and modified VSG-CRI. There was a notable stepwise increase in the risk of MACEs, from low to intermediate and to high intervals, within both the VSG-CRI and modified VSG-CRI risk classifications (*p* < 0.001, respectively). MACE, major adverse cardiac event, a composite endpoint of myocardial infarction, significant arrhythmias treated either by medical therapies or cardioversion, or congestive heart failure (CHF) during the hospital stay; VSG-CRI, Vascular Study Group Cardiac Risk Index. The Modified VSG-CRI (M VSG-CRI) additionally accounts for left ventricular wall motion abnormalities.

Patients were classified into three risk groups based on the VSG-CRI score for potential significant cardiac complications, including 134 (49.4%), 99 (36.5%), and 38 (14.0%) patients in the low (score 0–4), intermediate (score 5–6), and high (score > 6) risk groups, respectively. When divided based on the M VSG-CRI score, 196 (72.3%), 58 (21.4%), and 17 (6.3%) patients were at low (score 0–7), intermediate (score 8–9), and high (score > 9) risks, respectively (Figure 2).

The likelihood of developing MACEs increased correspondingly with higher VSG-CRI and M VSG-CRI scores. Compared to the VSG-CRI, the modified version demonstrated better MACE prediction for low-risk (15.3% vs. 11.9%), intermediate-risk (36.2% vs. 28.3%), and high-risk (58.8% vs. 44.7%) patients. The M VSG-CRI score detected more cardiac events at all risk levels than the VSG-CRI score (Figure 2).

## 4. Discussion

This study examined the role of LVWMAs in predicting MACEs among a diverse, real-world population requiring open abdominal aortic surgery. Nearly half of the patients displayed LWVMAs and reduced LVEF, reflecting the existing chronic cardiac and pulmonary conditions in this population. These findings underscore the difficulty in protecting these patients during major surgical interventions, given the increased likelihood of MACEs with higher cardiac risk scores. We identified LVWMAs as an additional independent predictor of MACEs. As a result, a modified risk score was developed that demonstrated better discriminatory capacity within our study population. With a C-statistic of 0.74 (95% CI 0.68–0.81), the modified risk score outperformed the conventional one, which had a C-statistic of 0.70 (95% CI 0.63–0.77). This reinforces the importance of assessing and properly addressing all known risk factors before performing major vascular surgical procedures.

Elective surgeries involving the abdominal aorta inherently carry higher periprocedural risks. The overall risk of patients is determined by both the inherent surgical risk and their specific health conditions, especially chronic coronary syndrome. Although extensive forms of CAD are common among patients with vascular diseases, it often remains asymptomatic (19). Silent ischemia, which can either pre-exist or emerge due to the stress of surgery, can lead to unfavorable clinical outcomes even in patients with a negative preoperative stress test (20). Some of these patients may exhibit LVWMAs before surgery, necessitating a patient-centered approach to their perioperative care. Notably, silent ischemia usually places these patients at a lower risk, according to the VSG-CRI classification, despite the higher risk of adverse events demonstrated in this study. In addition, surgical techniques, such as clamping and unclamping the aorta, significantly increase the workload on the LV due to changes in afterload, preload, as well as mean arterial and coronary perfusion pressure. These changes can affect the ejection fraction and induce wall motion abnormalities, possibly leading to adverse cardiac events (21). Thus, in alignment with recently released ESC Guidelines on the cardiovascular assessment and management of patients undergoing non-cardiac surgery (11), there is an urgent need to reassess and potentially enhance risk stratification methods by utilizing routine cardiac imaging tools that are widely available nowadays as a standard part of the risk stratification plan.

Although this subgroup was analyzed *ad-hoc*, a higher proportion of patients with comorbidities, such as CAD, was observed in the group of patients who experienced MACEs. This finding aligns with recent research demonstrating an association between the extent of CAD and the occurrence of MACEs (22). However, the treatment approach for these patient populations remains challenging. Despite the fact that numerous studies have examined the role of preoperative coronary revascularization in noncardiac surgery to reduce cardiac comorbidity and mortality (23, 24), the findings are conflicting. Furthermore, two distinct randomized trials failed to establish any advantage of preoperative revascularization in reducing postoperative myocardial infarction rates in major vascular surgery patients (25, 26). In this study, patients with a history of coronary revascularization, either with CABG or PCI, were also more frequently found in the group that experienced MACEs, nearly double the rate of those without any history of revascularization. Although prioritized coronary revascularization may lower the risk of MACEs in patients with specific aortic disease, it is noteworthy to emphasize the importance of optimal medical management in this setting. These include carefully titrated antihypertensive, lipid-lowering, and antithrombotic medical therapies, along with rigorous control of risk factors. This approach should be followed until robust evidence emerges to guide alternative treatment strategies. For patients identified with elevated cardiac risk, such as those with WMAs, the use of invasive hemodynamic monitoring methods like pulmonary artery catheterization and/or TEE can be critical, as the benefits often outweigh the procedure risks (27). These monitoring tools offer real-time insights into shifts in cardiac function and hemodynamic status. Such approaches, along with close collaboration between the vascular surgeon and anesthesiologist, are vital in effectively managing patients with complex aortic diseases, enabling personalized, condition-specific treatments.

In the face of challenges a single center may encounter in accumulating extensive cohort data from patients, the high prevalence of adverse events within the composite endpoint enables more reliable modeling, as evidenced by the narrow CIs (28). The incidence rate of MACE was 22.5%. A derivative study by Bertges et al. demonstrated a similar rate of composite cardiac outcomes in patients undergoing vascular surgery, with rates of 19.3% in the cohort derivation group and 22.6% in the validation group (8). The similarity in the MACE incidence could be attributed to extensive patient cohorts and the fact that all procedures were performed at tertiary health centers, which typically receive referrals for patients with more complex conditions. Importantly, the M VSG-CRI model, which integrates the VSG-CRI model and TTE parameters, showed enhanced performance compared to that of the standard VSG-CRI in predicting cardiac risk across low-, intermediate-, and high-risk patient groups. While the highest number of expected adverse cardiac outcomes is observed among high-risk patients, accurately identifying patients at cardiac risk who are initially classified as having a lower risk may have the most significant clinical relevance. This is particularly vital because these patients are often inadequately protected in the perioperative settings.

### 4.1. Strengths and limitations

A key strength of the present study lies in its contribution to better predictability of cardiac outcomes as a part of routine cardiac risk assessment without raising costs or delaying surgical treatment. The M VSG-CRI score is easily applicable and simple to calculate, which aids in preoperative, intraoperative, and postoperative management, as well as discharge medication and follow-up practice. It combines patient characteristics with preoperative TTE findings, a tool routinely used in preoperative assessment everywhere.

Despite these strengths, this research is subject to several limitations. The primary limitation of this study is the lack of an external validation group to evaluate the performance of the proposed modified scoring system. The fact that the data were collected from a single center and predominantly involved white European participants may limit the applicability of our findings to broader populations. Second, while our sample size was statistically projected 246 based on event proportion of 20% to secure a reliable 95% CI of 0.25–0.35, ensuring a maximum error of 0.05, the limited cohort size restrained our ability to examine essential subgroup differences effectively. Although we enrolled 10% more patients to mitigate potential reductions in sample size or protocol deviations, we did not encounter such issues. Still, this increase in sample size yielded only minimal statistical improvement, insufficient for any further statistical analysis. We could not draw definitive conclusions about the effects of reduced LVEF due to the relatively small number of patients in the present cohort. As a result, this factor wasn't incorporated into the newly derived model. Besides, our limited sample size also prevented us from distinguishing the influence of specific regional LVWMAs on the occurrence of MACEs, which could probably hold clinically meaningful value. Third, despite written intrahospital protocols for postoperative arrhythmia management, the potential for interobserver variability in assessment and clinical treatment cannot be completely ruled out. This inherent variability underscores the complexity of achieving a standardized approach in such cases. Lastly, the inclusion of endovascular cases in this patient cohort was also constrained by the financial limitations imposed by our public insurance system. Given budgetary constraints, major endovascular procedures are predominantly reserved for patients who are not deemed suitable for surgery. This circumstance significantly influenced our exclusion criteria, as it impeded our capacity to effectively adjust for patient characteristics between the open and endovascular repair procedures. This issue is further exacerbated by the limitations inherent in our data management system, which is not equipped to capture the full spectrum of details necessary to express varying degrees of patient frailty effectively.

## 5. Conclusion

Cardiac adverse events continue to be prevalent after open abdominal aortic surgery, mainly due to the substantial number of patients with concurrent cardiac conditions. The identification of LVWMAs through routine preoperative TTE holds significant predictive value and clinical implications, which importantly, can enhance the ability of cardiac risk score to predict major adverse events after abdominal aortic surgery. This insight could be instrumental in guiding clinicians during preoperative decision-making, particularly in identifying patients who may require meticulous perioperative care and cardiac risk reduction strategies. However, before this predictive model can be incorporated into daily clinical practice, it is essential to undertake further external validation to confirm its effectiveness across a wide range of patient populations.

## Data Availability

The raw data supporting the conclusions of this article will be made available by the authors, without undue reservation.
